# Large and giant intracranial aneurysms: outcomes from the multicenter prospective SMART coils registry

**DOI:** 10.3389/fneur.2025.1670622

**Published:** 2026-01-14

**Authors:** Yazan Ashouri, Alexandra R. Paul, Mohammad AlMajali, Amit Chaudhari, Eugene Lin, Min S. Park, Richard J. Bellon, Bradley N. Bohnstedt, Albert J. Yoo, Clemens M. Schirmer, Reade A. DeLeacy, David J. Fiorella, Keith Woodward, Harris E. Hawk, Ashish Nanda, Peter J. Sunenshine, Mouhammed R. Kabbani, Kenneth V. Snyder, Thinesh Sivapatham, Travis M. Dumont, Alan R. Reeves, Robert M. Starke, Alejandro M. Spiotta, Osama O. Zaidat

**Affiliations:** 1 St. Vincent Mercy Health Medical Center, Toledo, OH, United States; 2 Department of Neurosurgery, Albany Medical Center, Albany, NY, United States; 3 Department of Neurosurgery, University of Virginia Health, Charlottesville, VA, United States; 4 Department of Neurointerventional Surgery, Radiology Imaging Associates (RIA) Neurovascular, Englewood, CO, United States; 5 Department of Neurosurgery, Indiana University Health, Indianapolis, IN, United States; 6 Department of Interventional Neuroradiology, Texas Stroke Institute, Dallas, TX, United States; 7 Department of Neurological Surgery, Geisinger Medical Center, Danville, PA, United States; 8 Department of Neurosurgery, The Mount Sinai Hospital, New York, NY, United States; 9 Department of Neurosurgery, Stony Brook University Medical Center, Stony Brook, NY, United States; 10 Fort Sanders Regional Medical Center, Knoxville, TN, United States; 11 Department of Neurosurgery, Erlanger Health System Chattanooga, Chattanooga, TN, United States; 12 Providence St. Jude Medical Center, Fullerton, CA, United States; 13 Banner – University Medical Center Phoenix, Phoenix, AZ, United States; 14 Gundersen Health System, La Crosse, WI, United States; 15 Department of Neurosurgery, University of Buffalo, Buffalo, NY, United States; 16 Christiana Care Health System, Newark, DE, United States; 17 Department of Surgery, University of Arizona, Tucson, AZ, United States; 18 Department of Radiology, University of Kansas, Kansas City, KS, United States; 19 Department of Neurological Surgery, University of Miami Hospital, Miami, FL, United States; 20 Department of Neurosurgery, Medical University of South Carolina, Charleston, SC, United States

**Keywords:** endovascular treatment, coil embolization, large and giant aneurysms, intracranial aneurysm, intracranial lesions

## Abstract

**Background:**

Endovascular coiling for intracerebral aneurysms has been evolving. Yet, large and giant aneurysms (LAGA) remain a significant challenge in any treatment modality and carry high rates of morbidity and mortality.

**Method:**

The SMART registry, a prospective, multicenter site-adjudicated trial, was used to identify patients with LAGA (Sac 10–25 mm for large and >25 mm for giant) treated with the Penumbra SMART COIL (SMART) system and compare their outcomes to patients with smaller aneurysms (SA; Sac ≤ 10 mm). Aneurysm occlusion per Raymond-Roy (RROC) scale, recanalization, retreatment, mortality, and serious device-related adverse events (SAEs) were analyzed.

**Results:**

A total of 133/905 (14.7%) enrolled patients had LAGA with a mean (SD) aneurysm size of 13.7 (3.59) mm for LAGA and 5.8 (1.95) mm for SA. LAGA were more likely to be non-saccular (24.1% vs. 12.3%, *p* = 0.0007) and wide-necked (69.9% vs. 59.7%, *p* = 0.0268) compared to SA. Primary coiling was the main treatment modality for LAGA and SA (43.6% vs. 43.3%; *p* = 1.0). However, LAGA were more likely to be treated with flow diversion in addition to coiling (6.0% vs. 1.0%, *p* < 0.001). At 1-year follow-up: (i) RROC I-II was 82.7% in LAGA and 91.2% in SA; *p* = 0.0166, (ii) recanalization rates were 13.8% vs. 12.7%; *p* = 0.7417, and retreatment rates were 11.5% vs. 6.4%; *p* = 0.0648, for LAGA and SA, respectively, and (iii) all-cause mortality was 9.8% in LAGA vs. 4.7% SA; *p* = 0.0222. The device-related SAEs rates were low and comparable between the two study groups (4.5% vs. 4.1%; *p* = 0.8153).

**Conclusion:**

The SMART registry demonstrated that endovascular coiling can be feasible and safe in patients with LAGA, however randomized controlled studies are needed for comparative effectiveness.

**Clinical trial registration:**

https://clinicaltrials.gov/study/NCT02729740.

## 1 Introduction

Large and giant aneurysms (LAGA) are defined as aneurysms with at least one dimension measuring (>10–25 mm) and (>25 mm), respectively. These relatively uncommon lesions, representing about 7% of unruptured intracranial aneurysms (1), are characterized by progressive growth, thrombosis, and a high risk of rupture (2–4), and mortality (5). LAGA pose a significant challenge for treatment due to their size, association with wide neck morphology, and proximity to cranial nerves and the brain stem.

Active treatment aiming for LAGA occlusion, prevention of the aneurysmal rebleeding, relief of mass effect, and reduction of embolic complications is commonly pursued. Endovascular coiling for the treatment of cerebral aneurysms has been evolving since the results of the International Subarachnoid Aneurysm Trial (ISAT) and the BRAT (Barrow Ruptured Aneurysm Trial) were published (6, 7). Despite improving treatment strategies, the efficacy and safety of endovascular coiling of LAGA are still debated, with a high risk of recurrence and the necessity of further treatment commonly cited (4, 8–11).

The results of the SMART Registry were recently published (12). In this article, we describe a subgroup analysis comparing outcomes for LAGA and small aneurysms (SA) treated with the Penumbra SMART COIL (SMART) System, which includes SMART COIL, Penumbra Coil 400 (PC400), and Penumbra Occlusive Device (POD) indicated for endovascular embolization in the peripheral and cerebral vasculature.

## 2 Methods

### 2.1 Design

The SMART Study was a prospective, single-arm, post-market, multicenter registry of the SMART System (Penumbra, Inc.; ClinicalTrials.gov Identifier: NCT02729740). The registry was approved by the local Institutional Review Board (IRB) and Ethics Committee (EC) and conducted in accordance with relevant clinical research regulations. Written informed consent was provided by the study participant or their legally authorized representative (LAR). Emergent patients were enrolled after signing the consent within one calendar day after the procedure or if a LAR signed on their behalf. Penumbra, Inc., provided sponsor oversight of this trial.

### 2.2 Eligibility criteria, outcomes and data collection

Eligibility criteria, outcomes, and data collection have been described previously (12). Patients were excluded if SMART, PC400, or POD coils accounted for <75% of the total number of coils implanted, if their life expectancy was <1 year, if they were already enrolled in the SMART Registry, or if they were participating in other investigations that could confound results. All study procedures were completed per site’s standard of care. Adjunctive techniques and devices were permitted per study inclusion criteria. The aneurysm occlusion status was determined from cerebral angiograms obtained immediately and 1 year (± 6 months) postprocedure according to Raymond–Roy occlusion classification (RROC, class I: complete obliteration, class II: residual neck, class III: residual aneurysm) (13). For ruptured aneurysms, clinical grading of SAH was determined at admission, with no restriction on grade for enrollment, using the Hunt and Hess scale (14). Coil packing density was calculated by using either software calculators or by calculating aneurysm volume assuming an ellipsoid model and coil volume [*V* = *π* (*p*/2)^2^ × *L*], where *p* represents primary coil diameter, and *L* represents coil length. Packing density was not calculated for patients with deconstructive treatment, fusiform or dissecting aneurysms (12). Wide-necked aneurysms were defined as follows: a dome-to-neck ratio <2 or a neck width ≥4 mm.

The retreatment rate at 1 year and device-related serious adverse events (SAEs) within 24 h of the procedure were the primary efficacy and primary safety outcomes. The secondary outcome was the immediate adequate occlusion, defined as RR occlusion Class I or II. Other short-term follow-up outcomes included all periprocedural SAEs (SAEs within 24 h of the procedure), SAEs 24 h after the procedure, and all-cause mortality within 24 h. One-year outcomes included adequate occlusion, recanalization, modified Rankin Scale (mRS) 0 to 2, all-cause mortality, and SAEs.

### 2.3 Statistical analysis

We reported two-sided a 95% t confidence intervals (continuous data) or Wald asymptotic intervals (categorical data) were presented. Continuous variables were summarized with descriptive statistics [*n*, mean, standard deviation, median, and interquartile range (IQR)]. Frequency counts and percentages of subjects within each category were included for categorical data. A subgroup analysis was conducted on aneurysm size in the overall population and the wide-neck aneurysm subset. Two-sided 95% Wald asymptotic intervals for the difference in binomial proportions and two-sided *p* values based on Fisher’s Exact Test for categorical data, the *t*-test for continuous data, and the Wilcoxon rank-sum test using normal approximations for ordinal data were reported for the subgroup comparison. SAS 9.4 (SAS Institute) was used for statistical programming.

## 3 Results

### 3.1 Demographics

The SMART registry included 905 patients with intracranial aneurysms treated at 67 North American sites between June 2016 and August 2018. Of these, 14.7% (*N* = 133) had LAGA (Table 1). Patients with LAGA were older [mean (SD), 62.0 (13.4) vs. 59.4 (12.5), *p* = 0.0299] compared to patients with SA. Female patients were predominant, comprising 70.7% of LAGA and 75.4% of SA patients (*p* = 0.28). In both groups, more than half of the patients were hypertensive (57.9% vs. 62.4%, *p* = 0.3348) and one-third were current smokers (33.1% vs. 33.5%, *p* = 1.0) for LAGA and SA, respectively.

**Table 1:** Demographics and baseline characteristics of patients with small vs. large and giant aneurysms.

Baseline characteristics	Small aneurysms ≤10 mm (*N* = 772)	Large and giant aneurysms >10 mm (*N* = 133)	Difference SA-LAGA (95% CI)	*p*-value
Age (mean, SD), years	59.4 (12.47)	62.0 (13.39)	−2.6 (−4.9, −0.3)	0.0299
Age, range (min, max)	17.0, 93.0	20.0, 87.0	NA	NA
Race				
American Indian or Alaska Native	0.4% (3/772)	0.0%	0.4% (−0.1, 0.8%)	1.0000
Asian	0.8% (6/772)	0.0%	0.8% (0.2, 1.4%)	0.6003
Black or African American	4.4% (34/772)	3.0% (4/133)	1.4% (−1.8, 4.6%)	0.6396
White	26.4% (204/772)	23.3% (31/133)	3.1% (−4.7, 10.9%)	0.5207
Native Hawaiian or Other Pacific Islander	0.0%	0.0%	NA	NA
Other	1.3% (10/772)	5.3% (7/133)	−4.0% (−7.8, −0.1%)	0.0068
Ethnicity				
Hispanic or Latino	3.4% (26/772)	3.8% (5/133)	−0.4% (−3.9, 3.1%)	0.7963
Not Hispanic or Latino	29.9% (231/772)	27.8% (37/133)	2.1% (−6.2, 10.4%)	0.6813
Female	75.4% (582/772)	70.7% (94/133)	4.7% (−3.6, 13.0%)	0.2800
Comorbidities				
Hypertension	62.4% (482/772)	57.9% (77/133)	4.5% (−4.5, 13.6%)	0.3348
Diabetes mellitus	14.4% (111/772)	16.5% (22/133)	−2.2% (−8.9, 4.6%)	0.5086
Smoking	63.2% (488/772)	61.7% (82/133)	1.6% (−7.4, 10.5%)	0.7707
Former	28.8% (222/772)	27.8% (37/133)	0.9% (−7.3, 9.2%)	0.9173
Current	33.5% (259/772)	33.1% (44/133)	0.5% (−8.2, 9.1%)	1.0000
Unknown	0.9% (7/772)	0.8% (1/133)	0.2% (−1.5, 1.8%)	1.0000
Coronary artery disease	4.0% (31/772)	8.3% (11/133)	−4.3% (−9.1, 0.6%)	0.0424
Peripheral artery disease	1.3% (10/772)	2.3% (3/133)	−1.0% (−3.6, 1.7%)	0.4208
Hyperlipidemia	12.8% (99/772)	10.5% (14/133)	2.3% (−3.4, 8.0%)	0.5698
Aneurysm size, mean (SD), mm	5.8 (1.95)	13.7 (3.59)	−8.0 (−8.4, −7.6)	<0.0001
Size range (min, max), mm	1.0, 10.0	10.1, 29.0	NA	NA
Aneurysm location				
MCA	12.7% (98/772)	11.3% (15/133)	1.4% (−4.5, 7.3%)	0.7764
ACom	28.0% (216/772)	10.5% (14/133)	17.5% (11.4, 23.6%)	<0.0001
ICA terminus	3.1% (24/772)	3.0% (4/133)	0.1% (−3.0, 3.3%)	1.0000
Basilar tip	0.8% (6/772)	3.8% (5/133)	−3.0% (−6.3, 0.3%)	0.0140
PCom	17.5% (135/772)	20.3% (27/133)	−2.8% (−10.2, 4.5%)	0.4624
Pericallosal	3.0% (23/772)	0.8% (1/133)	2.2% (0.3, 4.1%)	0.2367
Cavernous	0.6% (5/772)	4.5% (6/133)	−3.9% (−7.4, −0.3%)	0.0022
Aneurysm shape				
Saccular	87.7% (677/772)	75.9% (101/133)	11.8% (4.1, 19.4%)	0.0007
Fusiform	1.4% (11/772)	8.3% (11/133)	−6.8% (−11.6, −2.1%)	<0.0001
Dissecting	2.3% (18/772)	3.8% (5/133)	−1.4% (−4.8, 2.0%)	0.3652
Other	8.0% (62/772)	12.0% (16/133)	−4.0% (−9.9, 1.9%)	0.1332
Wide neck	59.7% (461/772)	69.9% (93/133)	−10.2% (−18.7, −1.7%)	0.0268
Small neck	37.0% (286/772)	24.1% (32/133)	13.0% (5.0, 21.0%)	0.0042
Aneurysm status				
Ruptured	31.7% (245/772)	32.3% (43/133)	−0.6% (−9.2, 8.0%)	0.9198
Unruptured	68.3% (527/772)	67.7% (90/133)	0.6% (−8.0, 9.2%)	0.9198

ACom, anterior communicating artery; ICA, internal carotid artery; MCA, middle cerebral artery; PCom, posterior communicating artery.

Confidence intervals to reflect two-sided 95% *t* confidence intervals (continuous data) or Wald asymptotic confidence intervals (categorical data) for the difference in the groups. *p*-values are based on the two-sided *t*-test (continuous data), Wilcoxon rank-sum test (ordinal data), or Fisher’s Exact Test (categorical data), subject to the caveat that the study is not powered for subgroup comparisons.

### 3.2 Aneurysm characteristics

Aneurysm characteristics for LAGA and SA are outlined in Table 1. The mean (SD) aneurysm size was 13.7 (3.59) mm for LAGA and 5.8 (1.95) mm for SA (*p* value <0.0001). Almost one-third of all aneurysms were ruptured (32.3% for LAGA and 31.7% for SA; *p* = 0.9198). Of these, 36.6% LAGA and 44.6% SA had Hunt and Hess ≥3 (*p* = 0.3958). The most common location for LAGA was the posterior communicating artery (PCom) (20.3%), while the most common location for SA was the anterior communicating artery (ACom) (28%). LAGA were more likely to present at the basilar tip (3.8% vs. 0.8%, *p* = 0.0140) and the cavernous segment (4.5% vs. 0.6%, *p* = 0.0022) and less likely to present in the ACom (10.5% vs. 28.0%, *p* < 0.0001) compared to SA. In terms of morphological characteristics, LAGA were less likely to be saccular (75.9% vs. 87.7%, *p* = 0.0007), more likely to be fusiform (8.3% vs. 1.4%, p < 0.0001), and wide-necked (69.9% vs. 59.7%, *p* = 0.0268) compared to SA.

### 3.3 Aneurysm treatment

**Figure 1:**
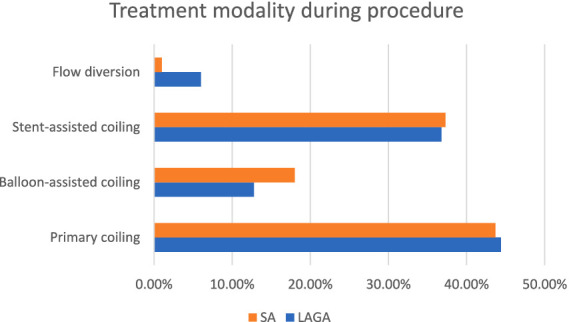
Endovascular treatment strategies employed in patients with small aneurysms (SA) vs. large and giant aneurysms (LAGA).

LAGA and SA were similar in the utilization of primary, unassisted coiling (43.6% vs. 43.3%), stent-assisted coiling (SAC, 36.8% vs. 37.3%), and balloon-assisted coiling (15.8% vs. 21.1%) (all *p* > 0.1) (Figure 1). However, LAGA were more likely to be treated with adjunctive flow diversion (6.0% vs. 1.0%, *p* = 0.0008) compared to SA. Within the LAGA group, a larger aneurysm sac was associated with lower rates of primary coiling (52.7% vs. 21.2% vs. 28.6%, *p* = 0.0028) and higher rates of flow diversion (2.2% vs. 12.1% vs. 28.6%, *p* = 0.0075) for aneurysms (10–15 mm), (15–20 mm) and (>20 mm), respectively. This trend was not seen in aneurysms <10 mm.

### 3.4 Treatment outcomes

Treatment outcomes for LAGA and SA are outlined in Table 2. Across the entire study cohort, the mean clinical and radiological follow-up period was 9.5 months (range: from 42 to 850 days). Packing density was significantly lower in LAGA, compared to SA [mean (SD) = 21.1(13.06) vs. 34.1(18.3), *p* < 0.001]. There was a trend toward lower packing density as aneurysmal size increase [Mean(SD) 23.5(18) vs. 17.9(10.6) vs. 13.3(8), *p* = 0.08] for aneurysm 10–15 mm vs. 15–20 vs. > 20. Adequate occlusion postprocedure (RROC I-II) was achieved in 58.1% of LAGA compared to 83.3% of SA, *p* < 0.0001.

**Table 2:** Clinical and angiographic outcomes in patients with small vs. large and giant aneurysms.

Treatment outcomes	Small aneurysms ≤10 mm (*N* = 772)	Large and giant aneurysms >10–25 mm (*N* = 133)	Difference SA-LAGA (95% CI)	*p*-value
Efficacy				
Packing density (calculated), mean (SD)%	34.1 (18.28)	21.1 (13.06)	13.0 (10.3, 15.8)	<0.0001
Raymond-Roy occlusion classification (postprocedure) %, (*n*/*N*)				<0.0001
Class I	43.8% (338/771)	23.3% (30/129)	20.6% (12.5, 28.7%)	
Class II	39.4% (304/771)	34.9% (45/129)	4.5% (−4.4, 13.5%)	
Class III	16.7% (129/771)	41.9% (54/129)	−25.1% (−34.0, −16.2%)	
Class I to II	83.3% (642/771)	58.1% (75/129)	25.1% (16.2, 34.0%)	<0.0001
Raymond-Roy occlusion classification (1-year follow-up)				0.0041
Class I	66.6% (409/614)	53.1% (52/98)	13.6% (3.0, 24.1%)	
Class II	24.6% (151/614)	29.6% (29/98)	−5.0% (−14.7, 4.7%)	
Class III	8.8% (54/614)	17.3% (17/98)	−8.6% (−16.4, −0.7%)	
Class I to II	91.2% (560/614)	82.7% (81/98)	8.6% (0.7, 16.4%)	0.0166
Aneurysm lesion occlusion % (*n*/*N*)^a^				
Better (progressive occlusion)	37.6% (231/614)	50.0% (47/94)	−12.4% (−23.2, −1.6%)	0.0237
Stable	49.7% (305/614)	36.2% (34/94)	13.5% (3.0, 24.0%)	0.0150
Worse (recanalization)	12.7% (78/614)	13.8% (13/94)	−1.1% (−8.6, 6.3%)	0.7417
Safety, % (*n*/*N*)				
Retreatment through follow-up% (*n*/*N*)	6.4% (40/627)	11.5% (12/104)	−5.2% (−11.6, 1.3%)	0.0648
Mortality, % (*n*/*N*)				
Within 24 h of procedure	0.1% (1/772)	0.0%	0.1% (−0.1, 0.4%)	1.0000
After 24 h from procedure	4.5% (35/772)	9.8% (13/133)	−5.2% (−10.5, 0.0%)	0.0197
Mortality at 1-year follow up	4.7% (36/772)	9.8% (13/133)	−5.1% (−10.4, 0.2%)	0.0222
Device-related mortality	0.1% (1/772)	0.0%	0.1% (−0.1, 0.4%)	1.0000
Procedure-related mortality	0.3% (2/772)	0.0%	0.3% (−0.1, 0.6%)	1.0000
Mortality after 365 days	0.3% (2/772)	1.5% (2/133)	−1.2% (−3.3, 0.9%)	0.1052
Serious adverse events, % (*n*/*N*)				
Device-related	4.1% (32/772)	4.5% (6/133)	−0.4% (−4.2, 3.4%)	0.8153
Procedure-related	8.7% (67/772)	9.8% (13/133)	−1.1% (6.5, 4.3%)	0.6236
Functional outcome				
mRS 0–2 at 1-year, % (*n*/*N*)	86.4% (376/435)	72.6% (53/73)	13.8% (3.1, 24.6%)	0.0047

a Calculated, compared to postprocedure.

“Not Applicable” and “Not Done” data are excluded from Raymond-Roy occlusion classification computations.

Confidence intervals to reflect two-sided 95% *t* confidence intervals (continuous data) or Wald asymptotic confidence intervals (categorical data) for the difference in the groups. *p*-Values are based on the two-sided *t*-test (continuous data), Wilcoxon rank-sum test (ordinal data), or Fisher’s Exact Test (categorical data), subject to the caveat that the study is not powered for subgroup comparisons.

Angiographic data at 1-year follow-up was available for 98 (73.7%) patients with LAGA and 614 (79.5%) patients with SA. Adequate occlusion (RROC I-II) on follow-up was lower in LAGA compared to SA (82.7% vs. 91.2%, *p* = 0.0166). However, the recanalization rates were similar between the two groups (13.8% vs. 12.7%, *p* = 0.7417 for LAGA and SA, respectively). Progressive occlusion of aneurysms on follow-up was more likely in patients with LAGA compared to SA (50.0% vs. 37.6%, *p* = 0.0237). Further, the retreatment rates through follow-up were similar between LAGA and SA (11.5% vs. 6.4%; *p* = 0.0648).

In terms of good functional outcome, LAGA patients were less likely to achieve mRS 0–2 at 1-year follow-up compared to SA patients (72.6% vs. 86.4%, *p* = 0.0047; Table 2). There was no device- or procedure-related periprocedural mortality in the LAGA cohort, while device- or procedure-related mortality was 0.3% in the SA cohort (*p* = 1.0). However, all-cause mortality >24 h postprocedure was significantly higher in LAGA (9.8% vs. 4.5%, *p* = 0.0197) compared to SA. Similar was true for all-cause mortality at 1-year follow-up (9.8% vs. 4.7% for LAGA and SA, respectively; *p* = 0.0222).

A subgroup analysis was performed to compare the treatment outcomes of patients with wide-neck LAGA and SA (Table 3). We noted that patients with wide-neck LAGA were less likely to achieve adequate occlusion postprocedure (RROC I-II: 53.8% vs. 80.7%, *p* < 0.0001) and at 1-year follow-up (82.1% vs. 93.7%, *p* = 0.0055) compared to wide-neck SA.

**Table 3:** Comparison of angiographic outcomes between patients with wide-neck small vs. wide-neck large and giant aneurysms.

Raymond-Roy occlusion	Wide neck ≤10 mm (*N* = 461)	Wide neck >10 mm (*N* = 93)	Difference SA-LAGA (95% CI)	*p*-value
Postprocedure				<0.0001
Class I	39.9% (184/461)	22.0% (20/91)	17.9% (8.3, 27.5%)	
Class II	40.8% (188/461)	31.9% (29/91)	8.9% (−1.7, 19.5%)	
Class III	19.3% (89/461)	46.2% (42/91)	−26.8% (−37.7%, −16.0%)	
Class I to II	80.7% (372/461)	53.8% (49/91)	26.8% (16.0, 37.7%)	<0.0001
Final follow-up				0.0002
Class I	69.6% (263/378)	47.8% (32/67)	21.8% (9.0, 34.6%)	
Class II	24.1% (91/378)	34.3% (23/67)	−10.3% (−22.4, 1.9%)	
Class III	6.3% (24/378)	17.9% (12/67)	−11.6% (−21.1%, −2.1%)	
Class I to II	93.7% (354/378)	82.1% (55/67)	11.6% (2.1, 21.1%)	0.0055

“Not Applicable” and “Not Done” data are excluded from Raymond-Roy occlusion classification (RROC) computations.

Confidence intervals to reflect two-sided 95% *t* confidence intervals (continuous data) or Wald asymptotic confidence intervals (categorical data) for the difference in the groups. *p*-Values are based on the two-sided *t*-test (continuous data), Wilcoxon rank-sum test (ordinal data), or Fisher’s Exact Test (categorical data), subject to the caveat that the study is not powered for subgroup comparisons.

Further analysis on adequate occlusion post-procedure and on follow-up revealed that BAC treatment showed a trend towards better adequate occlusion rates post-procedure(76.5%) with *p* = 0.07 compared to 60.3, 54.2 and 16.7% for primary coiling, SAC and flow diversion, respectively (Table 4). There was no statistical difference in terms of adequate occlusion on follow-up between different treatment techniques.

**Table 4:** Comparison of angiographic outcomes between different treatment approaches for patients with LAGA.

Raymond-Roy occlusion	Primary coiling	Balloon-assisted coiling	Stent-assisted coiling	Flow diversion	*p*-value
	*N* = 58	*N* = 17	*N* = 48	*N* = 6	
Postprocedure					0.112
Class I	17 (39.4%)	6(35.3%)	7(14.6%)	0	
Class II	18(31%)	7(41.2%)	19(39.6%)	1(16.7%)	
Class III	23(39.7%)	4(23.5%)	22(45.8%)	5(83.3%)	
Class I-II	35(60.3%)	11(76.5%)	26(54.2%)	1(16.7%)	0.071
Final follow-up	*N* = 45	*N* = 12	*N* = 34	*N* = 7	
Class I	23 (51.1%)	7 (58.3%)	18 (52.9%)	4 (57.1%)	1.00
Class II	14 (31.1%)	3 (25%)	19 (29.4%)	2 (28.6%)	
Class III	8 (17.8%)	2 (16.7%)	6 (17.6%)	1 (14.3%)	
Class I-II	37 (82.2%)	10 (83.3%)	28 (82.4%)	6 (85.7%)	0.966

A multivariate logistic regression model exploring predictive factors for 1-year adequate occlusion demonstrated that aneurysm size (aOR: 0.95, CI: 0.92–0.99, *p*: 0.005) was a significant predictor of achieving adequate occlusion on follow-up imaging. (Table 5).

**Table 5:** Multivariate logistic regression for predictors of 1-year adequate occlusion in patients with LAGA undergoing endovascular treatment.

Baseline characteristics	aOR	95% CI	*p*-value
Age	0.95	0.90–1.01	0.077
Packing density	1.00	0.99–1.01	0.249
Primary coiling	Ref	–	–
BAC	1.05	0.15–7.29	0.96
SAC	1.87	0.38–9.21	0.44
FD	11.2	0.5–253	0.13
Wide-neck aneurysm	2.31	0.54–9.94	0.260
Aneurysm type			
Saccular	Ref	–	–
Other	1.21	0.08–20.14	0.894
Fusiform	0.16	0.02–1.21	0.077
Dissecting	1.95	0.25–15.0	9,521
Aneurysm size (mm)	0.95	0.92–0.99	0.005
Ruptured aneurysm	1.08	0.26–4.40	0.918

aOR, adjusted odds ratio; BAC, balloon-assisted coiling; SAC, stent-assisted coiling; FD, flow-diverter; mm, millimeter.

Moreover, a multivariate analysis exploring factors associated with good functional outcome in patients with LAGA who had 1 year follow-up. After adjusting for possible confounders, aneurysm size and rupture status were associated with lower odds of achieving good functional outcome (Table 6). Dissecting aneurysms showed a trend towards worse functional outcome but did not reach statistical significance.

**Table 6:** Multivariate logistic regression for predictors of good functional outcome in patients with LAGA undergoing endovascular treatment.

Baseline characteristics	aOR	95% CI	*p*-value
Age	0.979	0.91–1.05	0.572
Packing density	1.04	0.99–1.01	0.149
Primary coiling	Ref	–	–
BAC	0.06	0.02–1.64	0.100
SAC	0.12	0.01–2.04	0.144
Wide-neck aneurysm	2.07	0.39–10.8	0.38
Aneurysm type			
Saccular	Ref	–	–
Fusiform	2.04	0.1–94.5	0.72
Dissecting	0.054	0.9–86.9	0.06
Aneurysm size (mm)	0.94	0.90–0.98	0.004
Ruptured aneurysm	0.029	0.01–0.414	0.009
Wide neck aneurysm	1.617	0.35–7.56	0.541

aOR, adjusted odds ratio; BAC, balloon-assisted coiling; SAC, stent-assisted coiling; FD, flow-diverter; mm, millimeter.

## 4 Discussion

Multiple series have reported safe outcomes in intracranial aneurysms treated using the SMART Coil system (15–18). The current subgroup analysis is the first to investigate the safety and efficacy of primary and assisted coiling in patients with LAGA treated with a single EVT coil family in an international, multicenter setting.

The stable embolization rate for LAGA using coils is typically lower than for SA (8). Patients in our series demonstrated similar occlusion rates and favorable outcomes to the previously published literature focused on endovascular coiling of LAGA. Immediate occlusion was found in 87.6% of aneurysms in the Chalouhi et al. (8), series and 58.1% in our series. At 1 year, an adequate occlusion was seen in 82.7% of cases, although our series had double the number of fusiform aneurysms (8.3% versus 4.1%).

Complication rates related to the target procedure were also similar; in our series 9.8% vs. 10.5% in Chalouhi et al. (8), and 10.2% in Wang et al. (19). Thirty-nine of aneurysms in the Chalouhi et al. (8) study demonstrated recanalization and 33% required further treatment by 25.4 months, compared to 13.8 and 11.5%, respectively, at 9.5 months in our series. This observation may (at least in part) be explained by a longer follow-up in the Chalouhi et al. (8) study compared to our series. Other groups have published recurrence rates as high as 57.9% with a retreatment rate of 25.5% (19).

The Chalouhi et al. (8) study also found that stent-assisted coiling was associated with lower recurrence rates and had 27.5% of cases in this category compared to 36.8% in our series. This aligns with the national trend moving away from primary coil embolization in more complex aneurysm cases (20).

Our all-cause mortality rate at 1 year (9.8%) was comparable to previous studies analyzing endovascularly treated large and giant aneurysms (19, 21, 22); importantly, none of the occurrences were device or procedure-related. One of the key findings of the study was the significant progression of occlusion in LAGA accompanied by a low recanalization rate. Notably, achieving RROC I-II in LAGA improved from 58.1% postprocedure to 82.7% on follow-up, and only 13.8% of aneurysms recanalized.

In summary, our series demonstrates that coil embolization of large and giant aneurysms with the SMART system yields occlusion rates and patient outcomes comparable to ones reported for other coiling technologies. These positive results may be partly due to the progressive coil softness and inner structural wire that might lead to less compartmentalization.

### 4.1 Limitations

This study followed an international, multicenter design that allowed for the evaluation of outcomes of the procedures conducted by different operators in various hospital care models. However, study limitations must be addressed to interpret our data fairly. The chief limitation is that data were provided by each study center without a core laboratory and blinded adjudication. As such, the physician-reported rates of aneurysm occlusion might have been subject to bias. The study was not powered for subgroup comparisons. Moreover, the utilization of dual antiplatelet agents was not collected and was at discretion of treating physician. Additionally, the primary intent of the study was to assess the outcomes of coiling technology. Alternative endovascular techniques, such as flow diverters, were out of scope for this analysis due to the limited data available. Finally, the study followed patients for 1 year only, and a longer-term follow-up would aid in understanding of post-treatment recurrence rates more accurately.

## 5 Conclusion

Despite the known challenges treating large and giant aneurysms, the SMART registry demonstrated high adequate aneurysm occlusion rates on follow-up imaging and good independent functional outcomes. This study provides further evidence for the efficiency and safety of the SMART System for the treatment of large and giant aneurysms in a real-world clinical setting.

## Data Availability

The datasets that support the findings of this study may be made available from the corresponding authors upon reasonable request. Requests to access the datasets should be directed to the corresponding author: yashouri.md@gmail.com.
